# Printed Nanocarbon Heaters for Stretchable Sport and Leisure Garments

**DOI:** 10.3390/ma15020573

**Published:** 2022-01-13

**Authors:** Andrew Claypole, James Claypole, Neil Bezodis, Liam Kilduff, David Gethin, Tim Claypole

**Affiliations:** 1 Welsh Centre for Printing and Coating, College of Engineering, Bay Campus, Swansea University, Swansea SA1 8EN, UK; andrew.claypole@swansea.ac.uk (A.C.); j.m.claypole@swansea.ac.uk (J.C.); d.t.gethin@swansea.ac.uk (D.G.); 2 Applied Sports, Technology, Exercise and Medicine (A-STEM) Research Centre, Swansea University, Swansea SA1 8EN, UK; n.e.bezodis@swansea.ac.uk (N.B.); l.kilduff@swansea.ac.uk (L.K.); 3 Welsh Institute of Performance Science (WIPS), Swansea University, Swansea SA1 8EN, UK

**Keywords:** Nanocarbon ink, printed heater, wearable, flexible, stretchable

## Abstract

The ability to maintain body temperature has been shown to bring about improvements in sporting performance. However, current solutions are limited with regards to flexibility, heating uniformity and robustness. An innovative screen-printed Nanocarbon heater is demonstrated which is robust to bending, folding, tensile extensions of up to 20% and machine washing. This combination of ink and substrate enables the heated garments to safely flex without impeding the wearer. It is capable of producing uniform heating over a 15 × 4 cm area using a conductive ink based on a blend of Graphite Nanoplatelets and Carbon Black. This can be attributed to the low roughness of the conductive carbon coating, the uniform distribution and good interconnection of the carbon particles. The heaters have a low thermal inertia, producing a rapid temperature response at low voltages, reaching equilibrium temperatures within 120 s of being switched on. The heaters reached the 40 °C required for wearable heating applications within 20 s at 12 Volts. Screen printing was demonstrated to be an effective method of controlling the printed layer thickness with good interlayer adhesion and contact for multiple printed layers. This can be used to regulate their electrical properties and hence adjust the heater performance.

## 1. Introduction

Flexible electrothermal heaters can be integrated into clothing to create wearable warming garments [1,2,3] and the ability to maintain body temperature has been shown to bring about improvements in sporting performance [4,5]. For example, using wire heated clothing at 40–42 °C during a post warm-up period of inactivity reduced losses in muscle temperature which was then associated with a performance improvement in sprint cycling [4]. Research has shown that a heater temperature of 40–43 °C should be considered a suitable target for heated clothing for elite sport [4,6]. Good control of heater temperature is essential for wearable applications, with the risk of skin burns above 45 °C [6]. Although wire-heating elements reduced losses in thigh muscle temperature, they were unable to fully attenuate them [4] and furthermore displayed a lack of temperature uniformity. The wire heated trousers were limited to 40 °C as activities such as sitting on a chair caused local increases in temperature with potential for burns [6]. The ability to produce uniform temperatures even when folded and flexed is of high importance. The effect of using higher heated garment temperatures was examined by using water perfused trousers at 43 °C and this virtually eliminated the drop in thigh muscle temperature during a 15-min period of inactivity, proving optimised heating will maintain muscle temperature. However, the water-perfused heating system was not suitable for use in the field [6]. The available wearable heating technology based on metal wire heaters have poor flexibility, suffer from oxidative corrosion, provide non-uniform heating, have low heating efficiency, limited heating areas and have a short lifetime on account of broken wires [1,2,7]. For wearable heaters to be used in sport, leisure or work activities they must be lightweight, mechanically robust, durable and capable of withstanding bending and stretching whilst not impeding the wearer or the garments’ ability to conform to body curvatures [8,9,10]. The ability to stretch would further improve the conformity of the device with the body [11,12]. A minimum target for repetitive strain of stretchable devices of 15–20% is required so as not to hinder fit and flexibility [13].

The ability to print a large-area, stretchable and flexible nanocarbon heater using low-cost inks and scalable techniques to create a wearable heating garment would be a disruptive technology. The ultimate objective of this research was to create a printed nanocarbon heater capable of achieving temperatures of 40 °C and to maintain a uniform temperature output when flexed and stretched. Delivery of this objective has required the development of carbon inks that possess appropriate mechanical and electrical properties together with their processability will be considered in the following paragraphs.

Printing is a scalable high throughput processes capable of producing thin, more flexible and stretchable devices with reduced production costs [11,12,13,14,15]. Polymer composites can be formulated into intrinsically flexible inks by dispersing conductive fillers within an elastic polymer matrix and a solvent, with electrical conductivity tuned by varying the loading of the filler material [8]. Flexible printed heaters have been constructed from inks containing graphene [7,16], carbon black [12], as well as hybrids of laser reduced graphene oxide and silver particles [2] and graphite and amino functionalised carbon nanotubes [1]. Metal nanostructure heaters can be operated at low driving voltages as they have low sheet resistance. However, oxidation at the junction between nanowires at high temperatures and their high material costs limit their practical use [2]. Polymer composites containing carbon nanofillers for electrical heating applications have advantages such as low weight, corrosion resistance, simple processing techniques, disposability, chemical inertness, controllable electronic properties and lower manufacturing costs [7,8,17,18,19]. Graphene based heaters often show fast heating rates and homogenous temperature distribution [16]. However, graphene-based heaters typically have large sheet resistance, and therefore require high input voltages for applications where higher temperatures are required which poses a potential injury risk. Therefore, a low resistance heater running at low voltages is preferable [2]. Printed heaters have used small carbon heater blocks between silver conductors to negate the high sheet resistance of carbon films [20]. The resistance of GNP only inks is also too high to be used in wearable heating applications [21] and printed heaters have used rigid substrates [1,2,7,16]. Although flex testing has been performed, the performance of the heaters under tensile extension was not typically studied. Commercially available carbon inks have also been shown to have insufficient electrothermal performance and poor durability with regards to creasing [2].

The authors have previously reported a stretchable screen printable nanocarbon ink with a printed sheet resistance of 230 Ω/□ capable of withstanding strains of >100% and cyclic strains of 10 and 100% [22]. This paper examines the viability of using this high conductivity stretchable nanocarbon ink to create lightweight, printed large area, heaters for use in wearable technology.

Screen printing allows for accurate patterning of thin films onto a wide variety of substrate materials [23]. The thickness of ink deposited is a function of the open screen area and the stencil thickness [24] as well as the solids loading, material and rheological properties of the ink [21,23]. The flow of ink through the mesh determines the uniformity of the printed surface [24], while the surface roughness is a function of the printing process, ink rheology and roughness due to the size and morphology of particles [17,23,25]. Particle surface topography and size variations provide a significant barrier to efficient charge transfer, which impacts on the electrical properties making it an important feature for conductive circuits [23,24,25]. In multi-layer devices the incumbent surface topography also influences the lay-down of subsequent layers, with the potential to impact overall device structure [23]. The printed heaters are multi-layer devices so the interaction between the carbon heater element and the subsequent carbon and silver layers used to deliver power to the heaters is of high importance.

A large-area, screen-printed nanocarbon heater is demonstrated using a highly conductive nanocarbon ink, capable of producing the 40 °C required for wearable heating, while showing good robustness to flexing, compressing, tensile extensions of up to 20% and machine washing. The heater can run from the low voltages required for use in wearable heating devices. Controlling the thickness of the printed layer through screen printing is shown to be an effective way of controlling the electrical properties of the printed heaters and their subsequent heater performance.

From a review of previous research, the challenges that are present in the printing of large area heaters for wearables comprise:

- The areas over which the power is applied, and the temperature measured.

These vary greatly from study to study. Lin, et al., produced a flexible heater by drop casting silver doped LRGO that could achieve 70 °C from a 15 V power supply over an approximately 4 cm^2^ area [2]. Park, et al., hand screen printed a graphite/amino functionalised CNT ink onto PET that could achieve 60 °C from a 12 V power supply [1]. Kang, et al., transferred CVD graphene onto PET to create a 16 cm^2^ heater that could achieve approximately 100 °C from 12 V [16]. An and Jeong solution cast a graphene epoxy composite onto PI film which could reach 126 °C at 30 V [7]. Pahalagedara, et al., screen printed a 12 cm^2^ CB/PU directly onto fabric that was capable of 65 °C from 15 V applied voltage [12].

- Exploration of film thickness and its impact on heater performance.

Increasing the number of printing carbon layers, and hence the print thickness decreases the sheet resistance of carbon prints [3,9,25]. Previous work has shown the effect of carbon layers to affect the temperature output of printed heaters [3]. For Joule heating devices changes to the resistance of the material would be expected to affect the heat output, however, the effect of heated film thickness on final heater output is often not studied.

- Determination of heater temperature uniformity.

Carbon and Silver/Carbon composite plane heaters have previously shown good heat uniformity [1,2,16]. Printed Graphite/Carbon Nanotube plane heaters consisting of carbon blocks between silver electrodes have been shown to have better uniformity than copper wire heaters, upon the application of voltage to the wire heaters only the wire heated up giving poor uniformity, while with the plane heaters both the silver tracks and carbon layers to give a more uniform heat. However, thermal images show that silver electrode structure still influences the heat uniformity. When the heaters were built into a car seat heater hot spots and large temperature variations across the whole heater are still visible [1]. Wearable silver wire heaters also show relatively poor uniformity compared to carbon plane heaters as the silver wire is the only part of the construction that heats [26].

- Heater performance when subject to creasing and strains up to 20%.

Printed heaters found in the literature have also been largely printed onto rigid films such as polyimide (PI) [2,7] and Polyethylene terephthalate (PET) [1,16] rather than softer more stretchable substrates such as thermoplastic polyurethane (TPU) or TPU blended substrates. Although bend testing has been performed, the performance of the heaters under tensile extension was not typically studied owing to the rigid nature of the substrates. Commercially available carbon inks have also been shown to have poor durability with regards to creasing [2]. This paper looks to demonstrate a lightweight, printed nanocarbon heater that shows good robustness to flexing, creasing and tensile strains to 20% nominal strain.

- The impact of washing cycles on heater durability and performance.

The ability to withstand several machine washing and wearing cycles are highly important for inks that are to be used with textile substrates [9], however, performance testing of wearable heating devices is often not carried out. Machine washing of PU/CB/Fabric heaters caused an increase in resistance of 14 ± 2% after the first wash, then increases of 2 ± 0.5% after the second cycle, however, the effect of this on the heating performance was not studied [12].

## 2. Materials and Methods

**Figure 1:**
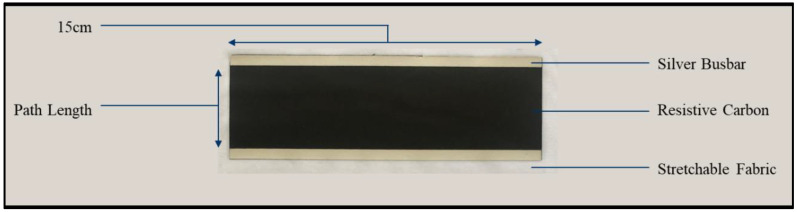
Annotated photograph of the screen-printed heater ironed onto a stretchable fabric base-layer.

Carbon and silver inks were used to fabricate the heater as shown in Figure 1. The carbon ink contained a blend of plasma functionalised Graphite Nanoplatelets (GNP) and Carbon Black (CB) in a commercially available Thermoplastic Polyurethane (TPU) resin in Diacetone alcohol (now commercialised as Flex Carbon C0001, Haydale Graphene ltd, Ammanford, UK). The silver ink used for the busbars consisted of 55 wt% of 10 μm silver flake (Silver flake 092, Technic, Cranston, RI, USA) in the same commercially available TPU and polar solvent. These inks were selected for their robustness to tensile strains as demonstrated in the authors previous work that describes the development of these inks [19].

To create the printed heaters firstly a large area 15 × 5 cm of the stretchable nanocarbon ink was screen-printed using a semi-automatic, flat-bed, screen press (DEK, ASM Assembly Systems GmbH & Co KG, Munich, Germany) with a 54–70 polyester mesh, 2.5 mm snap-off, a polyurethane diamond edge squeegee 130 mm length with a 12-kg squeegee force and print/flood speeds of 70 mm/s onto a commercially available, heat formable and stretchable PET/TPU substrate. Two 15 × 0.5 cm silver ink busbars were then printed on top of the carbon along parallel edges of the long side of the carbon block using the same print settings to create the printed heater (Figure 1). The effect of printing different numbers of layers of both the carbon and silver ink on heater performance was then studied. The screens also consisted of a 4.5 × 4.5 cm square to analyse the thickness, roughness and conductivity of the inks when printed. The inks were dried at 70 °C for 5 min. The inks were then heat pressed at 150 °C and 5 bars for 15 s using a tee-shirt heat press onto a stretchable base-layer fabric.

To calculate the print thickness and roughness white light interferometry with a 5× Lens and a 0.5% field of view to give an effective magnification of 2.5× was used to take 4 measurements from the edges of the printed squares from 5 separate samples. To calculate the sheet resistance of the prints a four-point probe with a 1.3 mm tip distance (SDKR-13, NAGY Messsysteme GmbH, Gäufelden, Germany) was used with a digital multimeter (Keithley, Tektronix, Beaverton, OR, USA). A correction factor of 4.5 was used as proposed by Smits [27]. To measure the two-point resistance of the inks two probes were used with the same digital multimeter.

To test the heater performance crocodile clips were used to connect opposing corners of the heaters to a controlled power supply (RS Components Ltd. Corby, Northants, UK). The heaters were suspended in mid-air to ensure no heat transfer to any other materials. The temperature of the heaters was then measured from the carbon area at the centre of the heaters using a thermal imaging camera (Optris PI precision line, Optris GmbH, Berlin, Germany) in a temperature-controlled laboratory (19 °C) using a figure of 0.98 for the emissivity of the carbon print [28]. The temperature response of the heaters was measured at voltages between 3–15 V applied for 180 s before also studying the temperature during cool-down for 60 s. The mean of three repeats of each condition was used with the standard deviation shown as error bars in the graphs.

**Figure 2:**
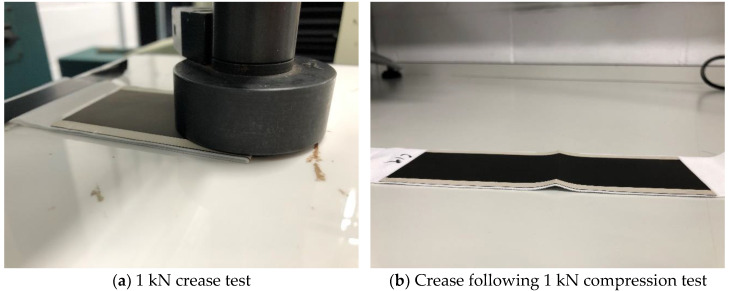
Photographs of (**a**) the compression being applied to the outside edge of the heater, (**b**) the crease following the compression.

To test for robustness, three heaters were subjected to a compression test, followed by an extension to fail test. To test the resistance of the heaters to creasing, 10 cycles of a 1 kN force was applied at a speed of 0.5 mm/min to the centre across the full 5 cm width of the heaters to create a permanent crease, with performance before and after creasing compared (Figure 2). To study the temperature performance of the heaters while under mechanical strains, two flat flex cable/flexible printed circuit connectors were attached to opposing corners of the heaters to give good mechanical connection to conventional wiring. A Hounsfield tensile tester (Hounsfield, Tinius Olsen TMC, Horsham, PA, USA) with a 10 kN load cell was then used to strain the heaters to strains of 0, 5, 10, 15, 20% to replicate the typical strains during wearing. The heaters were strained at a rate of 50 mm/min and held at each strain for 5 min. The heater performance was measured from an average of the temperature at minutes 3,4 and 5 at each relevant strain for three separate samples.

Having extended the three previous heaters used to failure, to examine the effect of machine washing on the heaters, three different heaters were passed through a commercially available domestic washing machine 5 times at 30 °C, 600 rpm, 15 mL biological washing detergent containing both anionic and non-ionic surfactants. The heaters were un-encapsulated to focus on the effect of machine washing on the inks. The heaters were allowed to air dry for 24 h before testing.

## 3. Results

A consistent ink that can be processed with scalable techniques is essential if mass production of printed designs is to be achieved. As the heaters work through Joule heating the electrical properties of the carbon and silver prints have a significant impact on final heater performance, with these electrical properties likely to be a function of the bulk ink properties, print thickness and print topography [23,24,25]. When screen printing functional inks for printed electronics it is important to control the thickness of the ink deposit as this will affect the prints electrical properties [29]. The carbon and silver inks had similar film layer thicknesses at 8.87 and 8.18 μm respectively (Table 1), irrespective of ink formulation, showing good agreement with the literature which states that the screen parameters, namely screen emulsion and the mesh are the major factors in controlling the print thickness [24,29].

The carbon inks have a significantly higher sheet resistance despite their similar thickness to the silver counterparts, because of the significantly higher bulk conductivity properties of silver. The sheet resistance of both the carbon and silver ink are in good agreement with the authors’ previous work demonstrating the repeatability of the ink production and the screen-printing process [22]. This allows for the design of a printed heater where the significantly more conductive silver ink is used to uniformly deliver current to the heat generating resistive carbon heater block.

**Figure 3:**
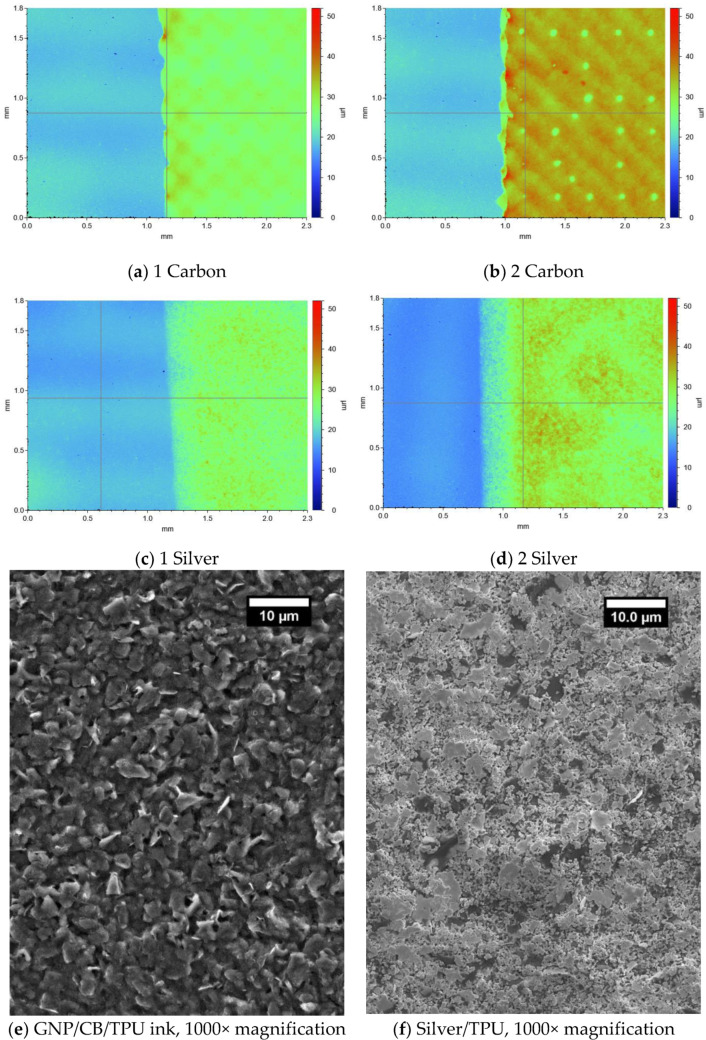
White light images at 2.5× magnification of the dry screen-printed coatings (**a**) Carbon, (**b**) 2-layer carbon, (**c**) Silver, (**d**) 2-layer silver, (**e**,**f**) Scanning electron microscope images of (**e**) GNP/CB ink at 1000×, (**f**) Silver flake ink at 1000× from [22].

The single layer nanocarbon print had a lower average surface roughness of 0.80 μm ± 0.19 (s.d) than the 1.29 μm ± 0.05 (s.d) single layer silver print. The white light images of single layer carbon (Figure 3a) and the single layer silver (Figure 3c) show no significant patterning on the surface of the coatings, suggesting that both inks were able to flow through the screen and relax to form a smooth coating, showing their suitability for screen printing. In a previous work by the authors Scanning Electron Microscope images to examine the microstructure of the coatings produced by the inks used in this study showed a near homogeneous surface, with the GNP flake size shown to be 4.05 ± 0.89 μm the smaller silver flakes appear to form a less homogenous microstructure, with voids present in the structure (Figure 3e,f) [22]. This relatively poorer particle distribution is a potential cause for the higher roughness in the silver print. The ability of the carbon ink to easily pass through the screen to form a homogenous electrically conductive heater coating (Figure 3a), while having a well dispersed and well interconnected carbon microstructure (Figure 3e), with good contact between neighbouring nano carbons, will allow for uniform current flow through the whole heater coating and therefore homogenous heating across the whole heater surface. The silver ink can also be screen printed to form a smooth continuous layer, and irrespective of the coating roughness, the significantly lower resistance of the silver ink would allow for the silver to be used to deliver current evenly across the heaters with minimum voltage drop.

As the heaters are a multi-layer printed device where the Joule heating power is directly related to the resistance, the interaction between subsequent printed layers will be an important factor in the final performance of the heaters. The effect of printing two layers of each ink was used to understand the effect of doubling the print thickness of each of the inks on the resistance and heater performance. Printing a second layer of functional material has previously been shown to decrease the resistance of carbon prints as the thickness of conductive material increases [9,25]. However, printing a second layer of conductive material does not always result in decreases in resistance. Previous research found increases in resistance when printing a second layer of silver onto paper. This was attributed to either the process of printing the second layer removing functional material, or the high resistance base in the second layer creating a barrier preventing the two layers interacting [30]. If there is good interaction between subsequent ink layers, then overprints could control the print thickness, and therefore the conductivity and heat output of the printed heaters. Printing a second layer of ink approximately doubled the print thickness of both the carbon and silver inks (Table 1). The two-layer carbon has higher roughness than the single layer carbon as a consequence of patterning in the print (Figure 3b). Irrespective of this patterning, printing a second layer of both the carbon and silver inks more than halved the sheet resistance of the coating, indicating that the increased thickness of the functional materials is the dominant factor in decreasing the resistance as a good interaction between the two subsequent print layers give a greater number of electronic pathways for the current to pass through.

**Figure 4:**
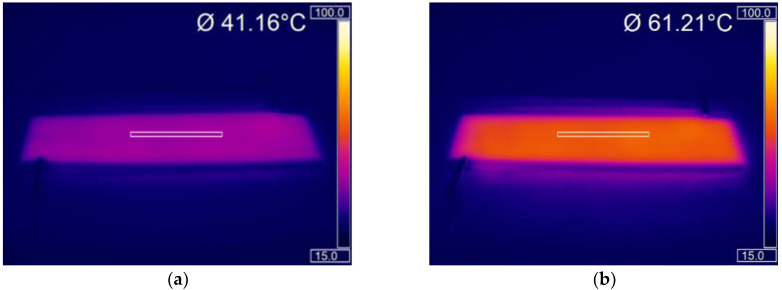

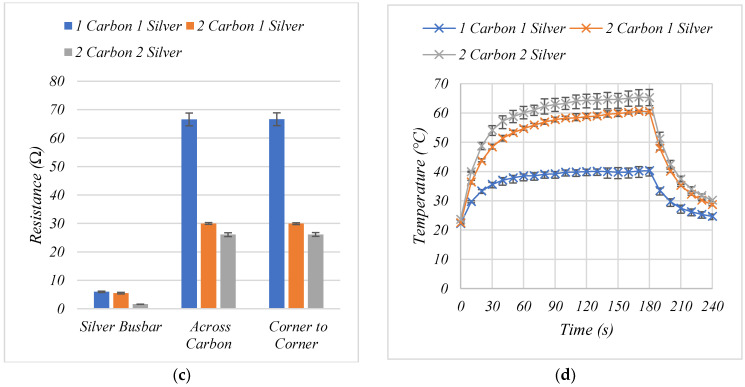
The effect of ink layering on the performance of single layer carbon and silver, double layer carbon and single layer silver and a double layer carbon and double layer silver heaters. Thermal image of (**a**) 1 Carbon 1 Silver heater, (**b**) 2 Carbon 1 Silver Heaters, (**c**) the mean two-point electrical resistance at 3 different points across the heaters from *n* = 5 samples with the error bars representing the standard deviation. (**d**) Average temperature-time performance of various heater constructions from *n* = 3 samples, with error bars showing the range of values.

The effect of printing various combinations of carbon and silver ink on the resistance along the silver busbar, from opposing corners and across the silver track and its subsequent effect on heater performance can be seen in Figure 4. The resistance of the heaters is equal across the carbon and from corner-to-corner for all the layering combinations demonstrating the effectiveness of the silver busbar in minimising voltage drop across the length of the heater (Figure 4c). This minimal voltage drop coupled with the ability of screen printing to accurately deposit smooth and thick layers of the highly conductive carbon ink results in a printed layer of uniform thickness and conductivity, capable of producing a highly uniform temperature distribution across the heaters (Figure 4a,b). This gives the printed heaters an advantage over conventional wire-based heaters, which show non-uniform heat output [1].

The effect of changing the ink layering structure on the heating performance was then examined on three heaters. For the three heaters tested printing a second layer of carbon more than halved the corner-to-corner resistance of the heaters from 54.07 Ω ± 2.87 (s.d) to 24.64 Ω ± 0.49 (s.d), this therefore more than doubled the joule heating power, with this increase in heating power associated with a faster heat up rate and a 20 °C increase in the temperature of the heaters to 60.45 °C within 180 s of being switched on (Figure 4d). Despite the presence of microscopic patterning in the surface of the two-layer carbon print (Figure 3b) the two-layer carbon heater still shows good temperature output uniformity (Figure 4b). This indicates that minor surface patterning doesn’t affect the macroscopic temperature output, which could indicate the ability of the carbon layer to spread heat to less uniform areas of the coating. Printing a second layer of silver further decreased the resistance of the heaters from 24.64 Ω ± 0.49 (s.d) to 23.22 Ω ± 0.54 (s.d). However, this smaller decrease in resistance was associated with a proportionally smaller 5 °C increase in heating temperature to 65.24 °C, the reduced losses along the busbars maximising the power to the heaters.

Increasing the number of printed layers of carbon, and therefore the print thickness, decreased the electrical resistance and increased the heating power for a set voltage and the increase in electrical current decreases the working time for a fixed battery capacity. Therefore, as the heaters were already capable of achieving 65.2 °C from 15 V, the effect of printing greater than two carbon layers was not studied as it was hypothesised this would further decrease the resistance and subsequently increase the current demands of any final device. At an applied voltage of 15 V, irrespective of the number of carbon or silver layers all the heaters reached >40 °C within 130 s of the power being switched on. The heaters also all cooled to <31 °C within a minute of the power being switched off. The fast heating response would enable the potential for the design of control electronics that could give close control over heater temperature, which is an important factor for wearable applications, particularly when the heaters are in close contact to the skin. The lowest resistance two carbon layer, two silver layer heater was then used for further testing as it was hypothesised that the strains incurred during washing and wearing would increase the resistance of the heaters and therefore decrease their temperature output.

**Figure 5:**
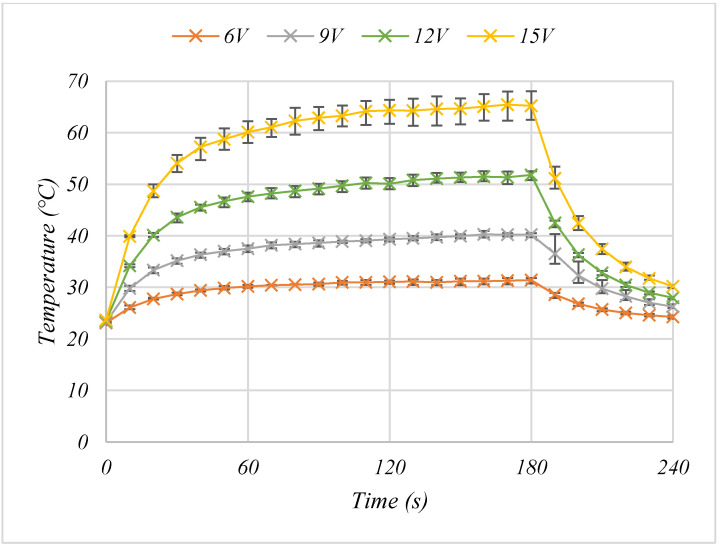
The effect of the applied voltage on the average temperature output of a 2-layer carbon, 2-layer silver heater where the values shown are the average of *n* = 3 samples with error bars showing the range of values.

The two layer carbon, two layer silver carbon achieved the highest temperature as a result of it having the lowest resistance, therefore, its heating performance was tested at voltages appropriate for wearable applications of between 6–15 V to identify the most suitable voltage for wearable heating applications (Figure 5). The heating power increased with applied voltage in accordance with Joule heating laws, with increased voltage also increasing the heat up rate. At 9 V the heaters could reach >40 °C within 160 s of switch on, at 12 V the heaters reached >40 °C within 20 s of switch on, before reaching a maximum temperature of 51 °C, while at 15 V they also achieved >40 °C within 20 s of switch on, before reaching a maximal temperature of 65 °C (Figure 5). Voltages of 14.8 V have previously been deemed suitable for wearable heating applications in sport [4], thus, that the heaters can heat to a temperature of 40 °C in a 19 °C environment from 15 V, shows the potential for use of these printed heaters in a practical elite sport setting without the requirement for larger, heavier power sources. The heaters show a rapid response, reaching near equilibrium temperatures within 120 s of the power being applied. Increasing the voltage increased the rate of heating with the 15, 12 and 9 V heaters reaching >39.88 °C within 10, 20 and 150 s, respectively. Therefore, where rapid heat up times are preferential, using a higher voltage to reach a set temperature and then limiting the voltage could be a prudent option. However, increasing the voltage also increases the power per area and therefore the size of the battery required for wearable applications. Thus, there is a trade-off between battery size and time between charging or replacing.

The heaters are an advance on printed heaters in the literature, especially when the area of the heater, 60 cm^2^, and the heat output is taken into consideration. Previous studies present the temperature rise for a given voltage over a small area, which is not an appropriate measure for effective large area wearable heaters.

The heaters consisting of two printed layers of carbon with two printed layer silver busbars and a voltage of 15 V was selected for further mechanical robustness testing as at 15 V the heaters reached temperatures >60 °C and showed a fast temperature response, heating to 48.7 °C within 20 s of switch on. Straining conductive inks has also been shown to increase the electrical resistance of conductive prints [22], therefore, it was hypothesised that under mechanical strain the resistance of the heaters would increase and the joule heating power of the heaters would decrease, decreasing the output temperature. It was hypothesised that by using heaters that could produce temperatures in excess of the 40 °C target set for wearable heating devices, this could account for any resistance increases incurred by mechanically straining the heaters, while the fast temperature response of the heaters could be used to design control electronics capable of controlling the temperature at 40 °C.

**Figure 6:**
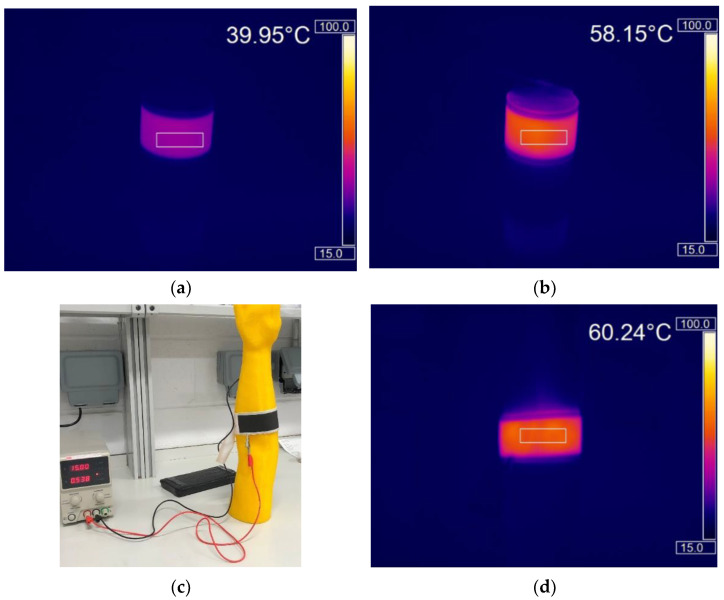
Two layer carbon, two layer silver heaters bent around various cylindrical objects. (**a**,**b**) Thermal images of the heaters after (**a**) 60 s and (**b**) at maximum temperature around a 6.5 cm diameter plastic pot. (**c**) Photograph and (**d**) thermal image of the heater tied around a 3D printed forearm.

To mimic the effect of being worn, the heaters were wrapped around both an 8 cm diameter plastic pot (Figure 6a,b) and a 3D printed arm (Figure 6c,d) and their heating performance monitored. The heaters still show good heat uniformity while bent and flexed making them appropriate for wearable heated clothing. This is a significant advantage over some of the current heated garments where hot spots formed while the garments were bent/stretched [6].

**Figure 7:**
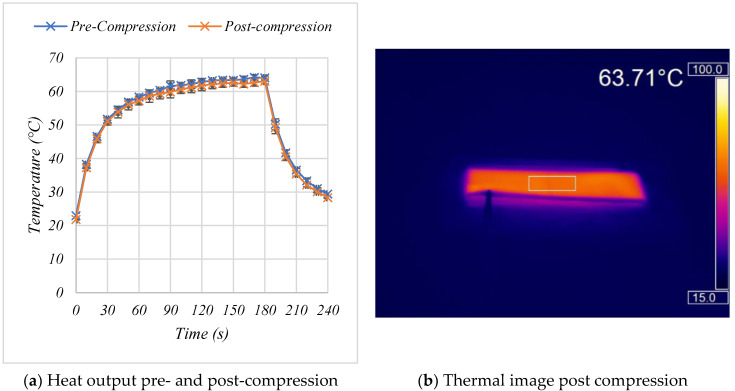
The effect of 1 kN compression on the performance of the two layer carbon, two layer silver heaters. (**a**) The effect of compression on the temperature-time response of the heater. (**b**) A thermal image showing the uniformity of heat output post compression.

To simulate creasing 10 cycles of a 1 kN force was applied to the outside edge of the heaters (Figure 2a). This force was selected to replicate human body weight and to put a permanent crease into the sample that would remain once the force had been removed (Figure 2b). Creasing of commercially available carbon inks [9] and silver inks has been shown to cause large increases in their electrical resistance [31] that would be expected to have a negative effect on the ability of the silver busbars to deliver power to the carbon heater elements and therefore heater performance. Following 10 creases there was no significant change in resistance as the heaters changed from 23.8 Ω ± 0.5 (s.d) to 23.7 Ω ± 0.5 (s.d). demonstrating the heaters good resistance to creasing. Following this permanent creasing the heaters still reached temperatures of 57.1, 61.7 and 63.0 °C at 60, 120 and 180 s following switch on, a decrease of 1.9, 1.8 and 1.6%, respectively, compared to their pre-compression values. Comparing the temperature achieved by the heaters before and after compression shows that the heater’s performance has not been affected with the heaters still capable of achieving 63 °C from 15 V, which is far greater than the 40 °C required for wearable heating applications (Figure 7a,b).

**Figure 8:**
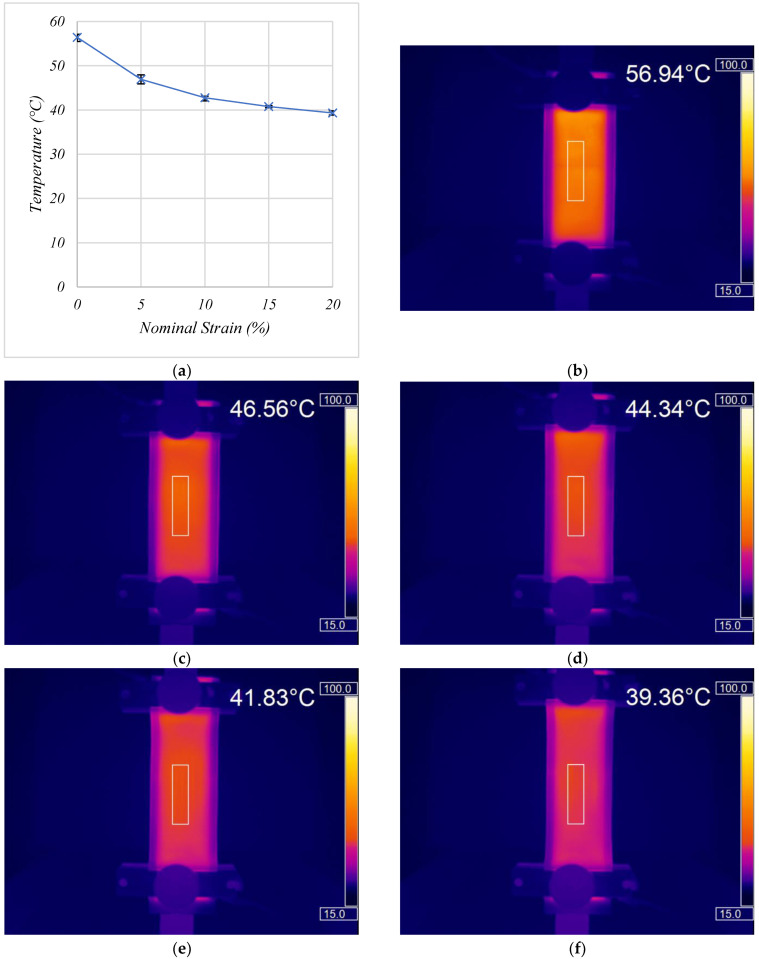
(**a**) The effect of nominal strain on the average heating temperature from *n* = 3 samples with error bars showing the range of values. Thermal images showing the effect of nominal strain on the uniformity of temperature output for a single heater at (**b**) 0%, (**c**) 5%, (**d**) 10%, (**e**) 15%, (**f**) 20% nominal strain.

At 0% nominal strain the heaters produce a uniform temperature of 56.4 °C (Figure 8a,b). This temperature is slightly lower than the 63 °C seen in the same heaters following the crease test which could be due to losses in the wired connections to the heaters for extension testing, increased air flow behind the suspended heaters and the heaters transferring heat to the Hounsfield grips. However, 56.4 °C is still greater than the target temperature of 40 °C. At 5% nominal strain, the heater temperature decreased to 46.9 °C suggesting a decrease in the Joule heating. The authors’ previous work demonstrated that applying tensile strains to the nanocarbon and silver inks increased their electrical resistance [22]. As Joule heating is inversely proportion to the resistance, this would bring about a decrease in the Joule heating power, while simultaneously increasing the heater area, that would be expected to be associated with a decrease in the output temperature.

Increasing the nominal strain to 10, 15 and 20% required maximum forces of 142, 156 and 173 N, respectively, and caused the temperature to decrease to 42.8, 40.8 and 39.3 °C, respectively (Figure 8a). The heaters maintained good heat uniformity and temperatures >40.7°C and >39.3 °C at nominal strains of 15 and 20% respectively, from a 15 V power supply, with no signs of hot spots that could be potentially dangerous to a human wearer. The good temperature uniformity under tensile strains suggests that good connection is maintained between neighbouring carbon particles even under tensile strains, allowing for even current flow across the coating and therefore even heating. The mechanism behind this maintained interparticle connection between the carbon particles even under tensile strains was suggested in a previous work by the authors using the inks used in this study, where the GNPs are decorated with the CB to form a well dispersed, dense 3D network with the larger GNPs lying at an angle to the substrate (Figure 3e,f). Under tensile strain the GNPs align in the direction of the strain, parallel to the substrate to maintain good interparticle contact [22]. The printed heaters maintained temperatures of >39 °C over a large area at nominal tensile strains of 20% and, following compressions of up to 1 kN. This demonstrates the robustness of the heaters to the distortion which could occur during rigorous physical activity and demonstrates their potential for safe, large area, wearable heated devices. The robustness of the heaters to strains of 20% (Figure 8), the fast temperature response and the ability to use voltage to control the temperature output of the heaters (Figure 5) could be used in combination with a simple microprocessor to maintain a uniform temperature output >39 °C between 0–20% nominal strain.

**Figure 9:**
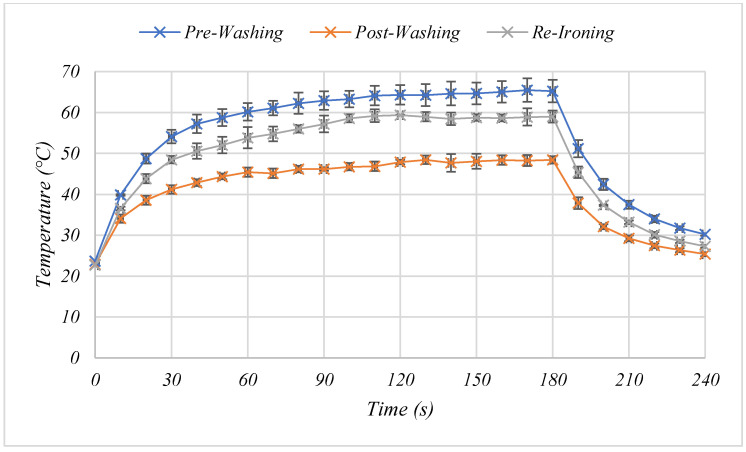
The effect of 5 cycles of machine-washing and re-ironing on the temperature-time relationship of a two layer carbon, two layer silver printed heater.

Being machine-washable is an important factor for the large-scale uptake of wearable technology. However, testing with regards to this often is not performed [9]. Following machine washing and air-drying the temperature output of the heaters dropped from 65.2 °C to 48.4 °C (Figure 9), likely as the resistance of the heaters increased from 22.94 Ω ± 0.44 (s.d) to 25.01 Ω ± 0.56 (s.d), potentially as a consequence of the substrate pulling away from the fabric, and the intense washing creating damage to the silver tracks. Machine washing of commercially available conductive carbon inks has previously been shown to cause resistance increases of >293%, while specially formulated carbon inks for textile printing have shown increases of >11.49% [9] and >14 ± 2% [12], therefore, the 8.28% increase shown by the inks used in this study following five washing cycles shows their good wash resistance. However, the heater performance recovered to 59 °C upon re-heat pressing as the resistance of the heaters returned to 22.56 Ω ± 0.69 (s.d). Willfahrt, Fischer and Hübner found that heat pressing decreased the electrical resistance, the roughness, and the thickness of screen-printed silver, as shrinkage of the binder allowed silver particles to have a better contact [31]. The recovery in performance following heat pressing could be a result of the application of heat and pressure softening the binder, allowing the silver flakes to re-flow and re-connect to repair any cracks or damage. Therefore, regular heat pressing could provide a means to repairing any damage that may occur through repeated machine washing. Further encapsulation would be expected to improve the heater adhesion to the fabric and to reduce some of the mechanical strain experienced by the heaters which is also likely to result in further improvements in wash resistance of the heaters.

## 4. Conclusions

Large-area carbon screen-printed heaters which utilise a blend of nano and micro carbons in a flexible resin have significant potential for wearable heating applications. The processability and bulk properties of the ink were investigated with screen-printing shown to be an efficient means of repeatedly producing and accurately depositing multiple layers of conductive inks over a large area for multi-layer devices. Interaction between multiple printed layers was studied, with good interconnection between subsequent printed layers. Printing a second carbon layer had the largest impact on the resistance and therefore, heater performance. Screen-printing process parameters can be used to control the thickness of the ink deposit, hence controlling electrical resistance and therefore heater performance.

The heaters showed a uniform temperature output over a large 15 × 4 cm area which can be attributed to the uniformity of the conductive carbon coating and the inherent thermal conductivity of the GNP. The heaters had a low thermal inertia, producing a rapid temperature response at low voltages, with the best heaters capable of reaching the 40 °C required for wearable heating applications within 20 s at 12 and 15 V. This rapid response to applied power should enable precise temperature control for the safe use of wearable heaters. At 15 V the heaters could reach a maximum temperature of 65.2 °C within 180 s of the power being switched on. The carbon heaters can reach the 40 °C required for wearable heating applications from a 9 V power supply albeit with slower response. This would allow a reduction in size of the required power supply for wearables, reducing the impact of any power supply on the comfort and portability of the garment.

The heaters demonstrated mechanical robustness with the heaters producing good temperature uniformity across the whole heated area while conformed around a variety of cylindrical objects and following a 1 kN crease test. Increases in the resistance of the heaters while in extension led to decreases in heater performance. However, the heaters continued to show good heat uniformity and could achieve temperatures in excess of 39.3 °C even while extended to 20% nominal strain, making them suitable for wearable heating applications. The heaters remained capable of heating to 48.4 °C after 5 washing cycles, with this performance further improved following re-ironing. These heaters were optimised and built into wearable garments used by British Athletes during training and in Tokyo in 2021 [32].

## Figures and Tables

**Table 1:** The print thickness and average surface roughness (Sa) from the screen-printed square from 20 measurements from *n* = 5 samples for the 1-layer carbon, 1-layer silver and 2-layers carbon and from 12 measurements from *n* = 3 samples for the 2-layer silver. The sheet resistance of the inks is shown for 28 measurements from *n* = 7 samples for the 1-layer carbon and 2-layer carbon, 56 measurements from *n* = 14 samples for the 1-layer silver and 24 measurements from *n* = 6 samples for the 2-layer silver.

	Thickness (μm)		Sa (μm)		Sheet Resistance (Ω∕□)	
	Average	SD	Average	SD	Average	SD
1 Carbon	8.87	0.72	0.80	0.19	226.13	4.31
2 Carbon	17.55	1.15	1.23	0.26	96.91	1.65
1 Silver	8.18	1.03	1.29	0.05	0.15	0.01
2 Silver	15.68	1.27	1.51	0.07	0.04	0.01

## Data Availability

Data is contained within the article.
